# Supplementary material for: Electron scale coherent structure as micro accelerator in the Earth’s magnetosheath
Source: Nat Commun. 2024 Jan 30;15:886. doi: 10.1038/s41467-024-45040-5 (PMC10824732; doi:10.1038/s41467-024-45040-5)
Supplement: Supplementary file 1 — Supplementary Information [file 41467_2024_45040_MOESM1_ESM.pdf]

Supplementary Information for

Electron Scale Coherent Structure as Micro Accelerator in the Earth's Magnetosheath

Zi-Kang Xie[1], Qiu-Gang Zong[1,2*], Chao Yue[1], Xu-Zhi Zhou[1], Zhi-Yang Liu[1], Jian-Sen He[1], Yi-Xin Hao[3], Chung-Sang Ng[4], Hui Zhang[5], Shu-Tao Yao[5], Craig Pollock[6], Guan Le[7], Robert Ergun[8], Per-Arne Lindqvist[9]

[1]Institute of Space Physics and Applied Technology, Peking University, Beijing 100871, China. [2]State Key Laboratory of Lunar and Planetary Sciences, Macau University of Science and Technology, Taipa, Macau, China. [3]Max Planck Institute for Solar System Research, Göttingen, Germany. [4]Geophysical Institute, University of Alaska Fairbanks, Fairbanks, AK, USA. [5]Shandong Provincial Key Laboratory of Optical Astronomy and Solar-Terrestrial Environment, Institute of Space Sciences, Shandong University, Weihai 264209, China. [6]Denali Scientific, 3771 Mariposa Lane, Fairbanks, AK 99709, USA. [7]Heliophysics Science Division, NASA, Goddard Space Flight Center, Greenbelt, MD 20771, USA. [8]Department of Astrophysical and Planetary Sciences, University of Colorado LASP, Boulder, CO, USA. [9]Department of Space and Plasma Physics, KTH Royal Institute of Technology, Stockholm, Sweden.

*Correspondence to: qgzong@pku.edu.cn

This PDF file includes:

Supplementary Figures 1 to 4

Supplementary Table 1

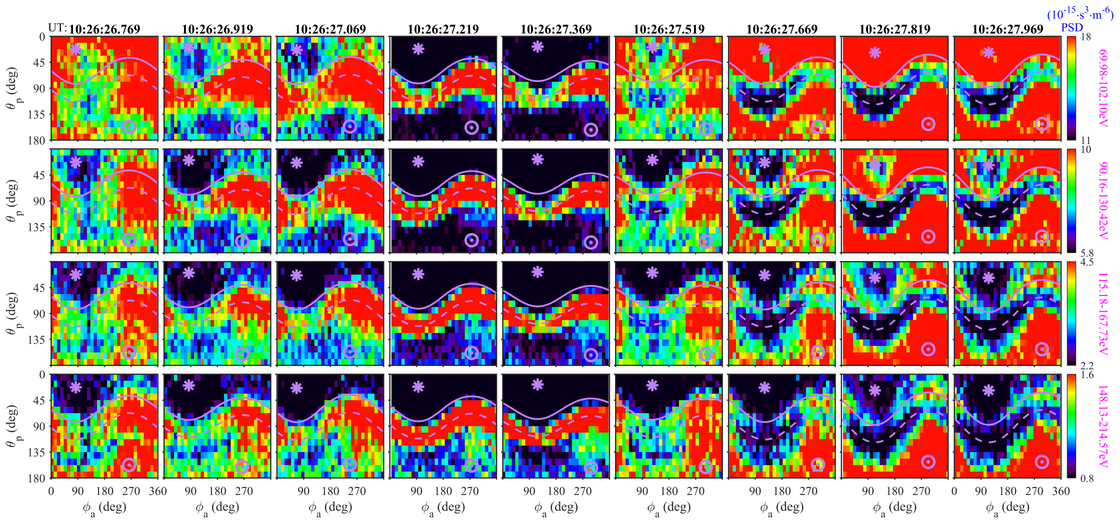

Supplementary Figure 1. Sky-maps for electron phase space densities inside the structure. Each column shows observations at different times, which are labeled on the top. Panels in the same row are from the same energy channel, and the energy range is labeled on the right. For each panel the horizontal and the vertical axes are azimuthal and polar angles in DBCS (De-spun Body Coordinate System), respectively. The asterisk and the circle represent parallel and anti-parallel directions to the magnetic field, respectively. In each panel, the dashed line represents 90° pitch angle in the sky-map, while the solid line is for 60° pitch angle.

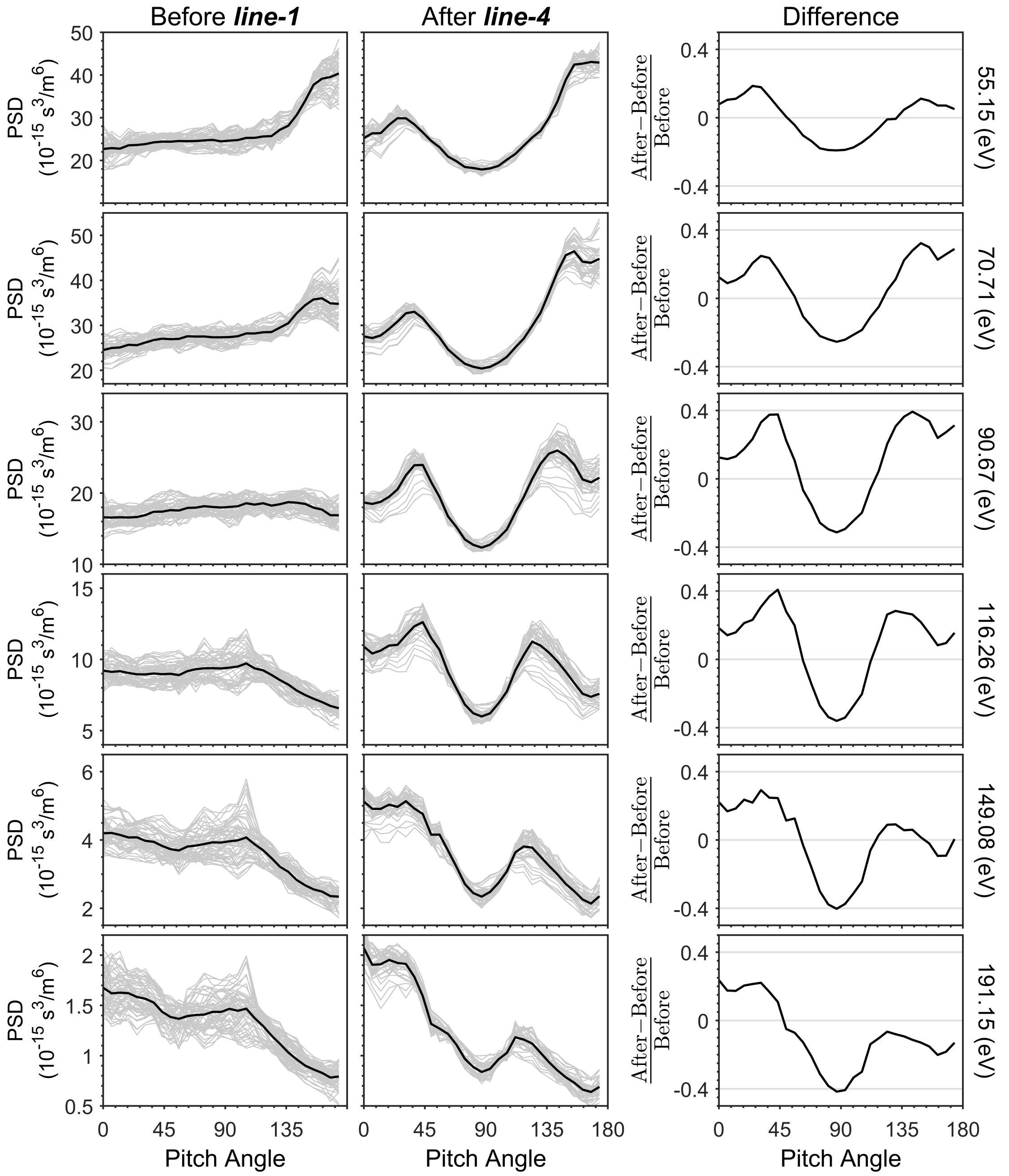

Supplementary Figure 2. Electron pitch angle distributions before line-1 and after line-4. From left to right, the first and second columns are the electron phase space density (PSD) before line-1 and after line-4, respectively (line-1 and line-4 are detailed in Figure 1 of the main text). The third column is the difference of the PSD relative to its value before line-1. Panels in the same row are from the same energy channel, and the energy is labeled on the right.

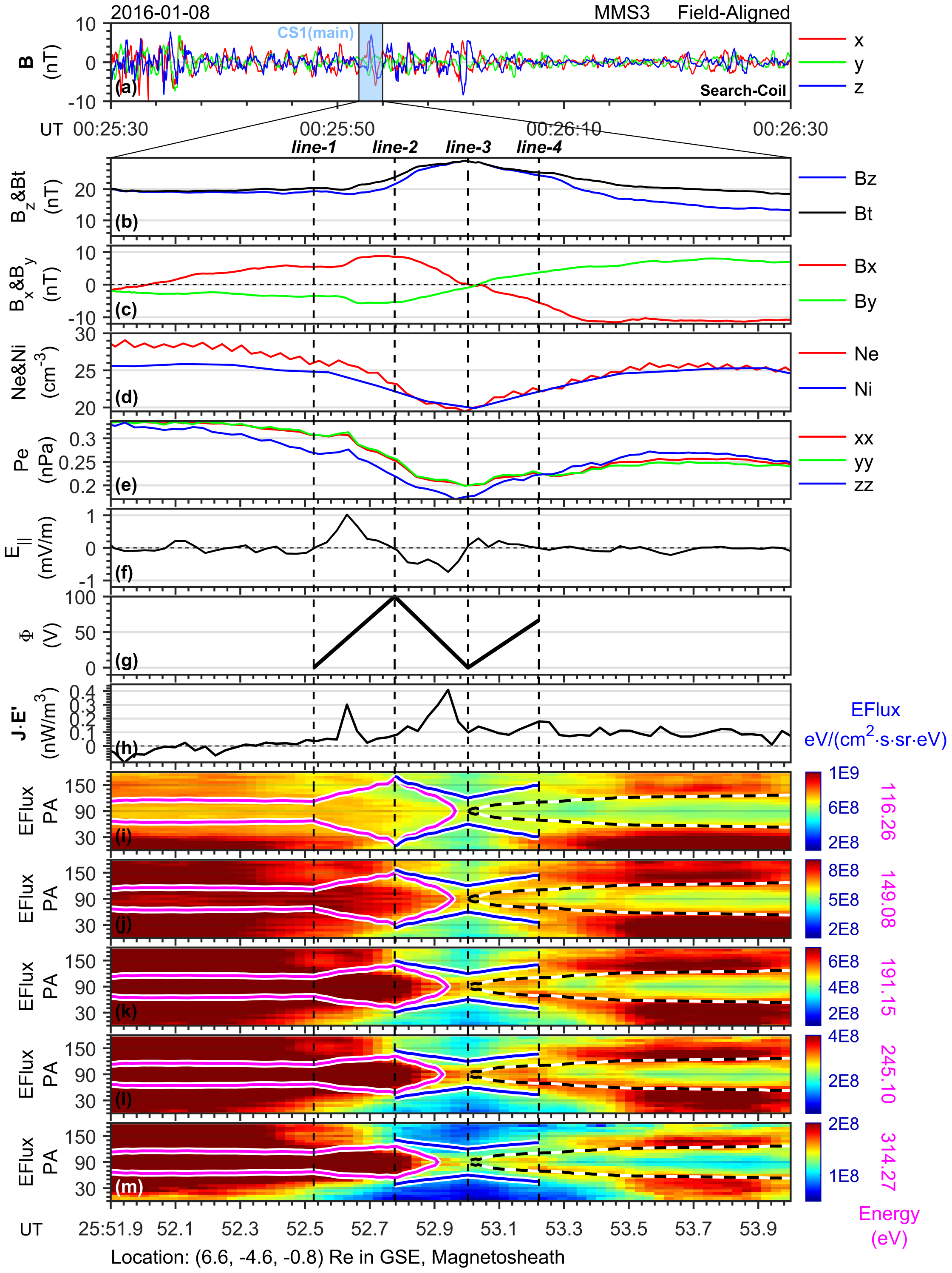

Supplementary Figure 3. Another coherent structure found in the turbulent magnetosheath. Panel (**a**) shows the turbulent magnetic fields at a 1-minute time scale, while the rest panels (**b**) – (**m**) are for only 2.1 seconds (CS1). Panels (**b**) and (**c**) show magnetic field components (B_x in red, B_y in green, B_z in blue, and the field strength

B_t in black) in the local field-aligned coordinates. Panels (**d**), (**e**), and (**f**) denote electron and ion number density, diagonal terms of electron pressure tensor, and parallel electric field ($E_{\|}$), respectively. Panel (**g**) shows the assumed electric potential. Panel (**h**) shows the $\mathbf{J} \cdot \mathbf{E}'$. Panels (**i**) - (**m**) show pitch angle distributions of electron energy flux of energies from ~116 eV to ~314 eV. There are four vertical dashed lines across all panels, where line-1 represents the begin of non-zero $E_{\|}$; line-2 and line-3 mark out the time when the sign of $E_{\|}$ is reversed; line-4 is the end of non-zero $E_{\|}$. In panels (**i**) - (**m**), the magenta lines represent the critical trapping angle α_l defined by Equation (6); the blue lines show the expected streaming region given an electron source at line-3 (60° to 120° pitch angle); the dashed black (white) lines reproduce single particle motion for an electron starting at line-3 (90° pitch angle) without the impact of $E_{\|}$. All the lines (magenta, blue, and dashed) are directly deduced by electromagnetic field observations (panels (**b**), (**c**), (**f**), and (**g**)). Spacecraft position is labeled at the bottom of the figure.

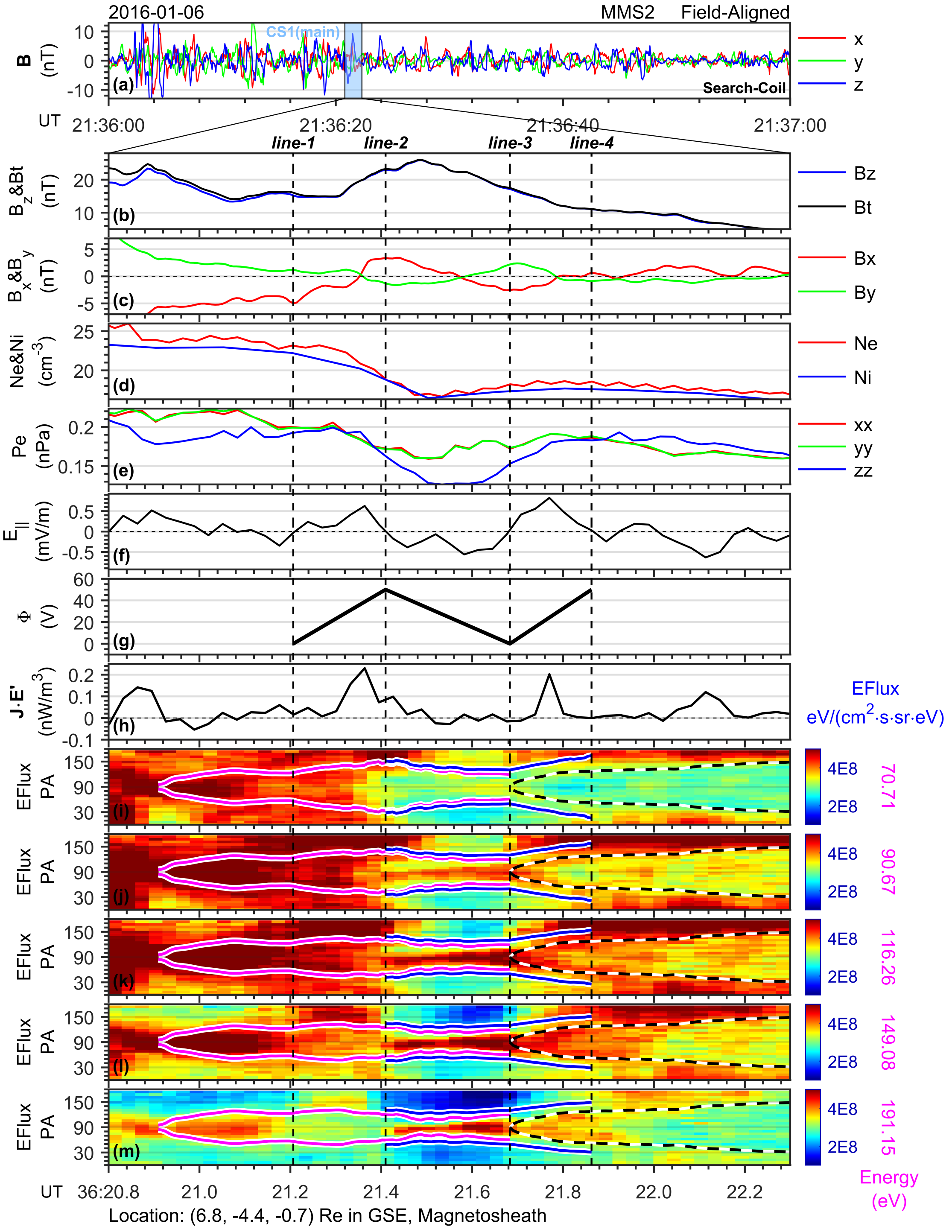

Supplementary Figure 4. Another coherent structure found in the turbulent magnetosheath. Panel (**a**) shows the turbulent magnetic fields at a 1-minute time scale, while the rest panels (**b**) – (**m**) are for only 1.5 seconds (CS1). Panels (**b**) and (**c**) show magnetic field components (B_x in red, B_y in green, B_z in blue, and the field strength

B_t in black) in the local field-aligned coordinates. Panels (**d**), (**e**), and (**f**) denote electron and ion number density, diagonal terms of electron pressure tensor, and parallel electric field ($E_{\|}$), respectively. Panel (**g**) shows the assumed electric potential. Panel (**h**) shows the $\mathbf{J} \cdot \mathbf{E}'$. Panels (**i**) - (**m**) show pitch angle distributions of electron energy flux of energies from ~70 eV to ~191 eV. There are four vertical dashed lines across all panels, where line-1 represents the begin of non-zero $E_{\|}$; line-2 and line-3 mark out the time when the sign of $E_{\|}$ is reversed; line-4 is the end of non-zero $E_{\|}$. In panels (**i**) - (**m**), the magenta lines represent the critical trapping angle α_l defined by Equation (6); the blue lines show the expected streaming region given an electron source at line-3 (60° to 120° pitch angle); the dashed black (white) lines reproduce single particle motion for an electron starting at line-3 (90° pitch angle) without the impact of $E_{\|}$. All the lines (magenta, blue, and dashed) are directly deduced by electromagnetic field observations (panels (**b**), (**c**), (**f**), and (**g**)). Spacecraft position is labeled at the bottom of the figure.

Supplementary Table 1. Start time of similar electron-scale structures, observed by MMS1. 54 coherent structures have been found from September 1 to 7, 2015.

2015-09-02T13:53:34UT	2015-09-02T17:02:44UT	2015-09-03T16:54:24UT
2015-09-02T15:27:24UT	2015-09-02T17:05:44UT	2015-09-03T16:54:34UT
2015-09-02T15:27:34UT	2015-09-02T17:13:54UT	2015-09-03T16:54:34UT
2015-09-02T15:27:44UT	2015-09-02T17:15:14UT	2015-09-03T16:55:24UT
2015-09-02T15:27:54UT	2015-09-02T17:16:24UT	2015-09-03T17:21:04UT
2015-09-02T15:28:44UT	2015-09-02T17:23:54UT	2015-09-03T17:21:54UT
2015-09-02T15:29:44UT	2015-09-02T17:30:24UT	2015-09-03T17:22:04UT
2015-09-02T15:29:54UT	2015-09-02T17:31:44UT	2015-09-03T17:22:24UT

2015-09-02T16:07:34UT	2015-09-03T14:24:24UT	2015-09-03T17:23:04UT
2015-09-02T16:11:04UT	2015-09-03T14:24:44UT	2015-09-03T17:23:14UT
2015-09-02T16:12:04UT	2015-09-03T14:31:04UT	2015-09-07T13:32:44UT
2015-09-02T16:13:24UT	2015-09-03T15:15:04UT	2015-09-07T13:33:24UT
2015-09-02T16:47:44UT	2015-09-03T16:14:44UT	2015-09-07T13:36:44UT
2015-09-02T16:56:14UT	2015-09-03T16:50:04UT	2015-09-07T13:38:24UT
2015-09-02T16:56:34UT	2015-09-03T16:52:54UT	2015-09-07T13:57:14UT
2015-09-02T17:00:34UT	2015-09-03T16:53:14UT	2015-09-07T13:58:44UT
2015-09-02T17:02:14UT	2015-09-03T16:54:04UT	2015-09-07T13:59:04UT
2015-09-02T17:02:24UT	2015-09-03T16:54:14UT	2015-09-07T13:59:54UT
